# Present and future structural biology activities at DESY and the European XFEL

**DOI:** 10.1107/S1600577525000669

**Published:** 2025-02-18

**Authors:** Dominik Oberthür, Johanna Hakanpää, Spyros Chatziefthymiou, Guilllaume Pompidor, Richard Bean, Henry N. Chapman, Edgar Weckert

**Affiliations:** a Deutsches Elektronen-Synchrotron DESY Notkestrasse 85 22607Hamburg Germany; b European XFEL Holzkoppel 4 22869Schenefeld Germany; c Centre for Ultrafast Imaging, Luruper Chausee 149, 22761Hamburg, Germany; d Department of Physics University of Hamburg Luruper Chausee 149 22761Hamburg Germany; Cornell University, USA

**Keywords:** structural biology, macromolecular crystallography, serial crystallography, FEL and synchrotron radiation

## Abstract

Structural biology investigations using synchrotron radiation have a long history at the photon science facilities at DESY. Presently, EMBL and DESY operate state-of-the-art macromolecular crystallography and biological SAXS stations at the synchrotron radiation source PETRA III for the international user community. New experimental opportunities for experiments with femtosecond temporal resolution and for extremely small macromolecular crystals have become available with the advent of X-ray free-electron lasers (XFELs) such as the European XFEL. Within large international collaborations, groups at DESY and the European XFEL have contributed significantly to the development of experimental and data analysis methods to enable serial crystallography experiments at both XFELs and high-brilliance synchrotron radiation sources. The available portfolio of analytical infrastructure in photon science at DESY has attracted several campus partners to contribute to the development of instruments and methods and provide their own complementary experimental techniques, thereby establishing a fruitful scientific environment to make significant contributions to present and future societal challenges in the field of life sciences.

## Abstract 2

Facilities for structural biology investigations at DESY and the European XFEL are described.

## 1. Introduction

The first scattering experiment from biological samples using synchrotron radiation was a small-angle X-ray scattering (SAXS) experiment from a muscle fibre carried out by Gerold Rosenbaum and Kenneth Holmes from the Max Planck Institute in Heidelberg, Germany, at the DESY synchrotron (Hamburg, Germany) in August 1970 (Rosenbaum *et al.*, 1971 ▸; Holmes & Rosenbaum, 1998 ▸). The results of these experiments made the potential of synchrotron radiation for life science applications immediately apparent, leading to the establishment of the European Molecular Biology Laboratory (EMBL) outstation at DESY in 1975. Later, several beamlines for structural biology were developed at the DORIS III storage ring. These included three beamlines for macromolecular X-ray crystallography (MX) and one for biological SAXS (bio-SAXS), all operated by EMBL, and one shared MX beamline operated by the Max Planck Society (MPG). During the transformation of the PETRA storage ring to a third-generation synchrotron radiation source, EMBL and MPG activities in the field of structural biology were transferred to the PETRA III facility, which resulted in dramatically improved beam parameters and experimental conditions with user operation on the first beamlines starting in 2010.

Presently, the DESY campus in Hamburg is home to three large-scale photon science facilities: the high-energy synchrotron radiation source PETRA III, the soft X-ray free-electron laser (XFEL) FLASH, and the injector complex of the 3 km-long European XFEL facility. With a circumference of 2304 km, PETRA III is the largest circumference storage ring-based X-ray source in the world. This third-generation source operates at 6 GeV with 120 mA beam current in quasi-continuous mode or with 100 mA in a timing mode with 192 ns bunch separation. The beam current is kept stable to within 1% by a top-up injection scheme. The facility provides 5000 h per year of X-ray beam to 25 beamlines with almost 60 experimental stations. Two additional beamlines are still under construction.

The beamlines at PETRA III are situated in three experimental halls. The largest is the 288 m-long Max von Laue experimental hall, which hosts 14 beamlines. The last two sectors of this experimental hall house the beamlines dedicated to biological samples, *i.e.* the MX beamlines P11 (Burkhardt *et al.*, 2016 ▸), P13 (Cianci *et al.*, 2017 ▸) and P14 (Albers *et al.*, 2024 ▸), as well as the bio-SAXS beamline P12 (Blanchet *et al.*, 2015 ▸). This last beamline benefits considerably from advanced SAXS analysis software that has been developed in-house at EMBL (Manalastas-Cantos *et al.*, 2021 ▸). Beamline P11^1^ is operated by DESY and beamlines P12–P14^2^ are operated by the EMBL. Beamline P14 also hosts a station for time-resolved macromolecular crystallography called T-REXX that is operated in collaboration with the University of Hamburg. In addition, P14 also provides sub-micrometre tomographic X-ray imaging of biological specimens. All beamlines deliver an Si(111) monochromatic flux of about 10^13^ photons s^−1^ in the unfocused beam and about 10^12^ photons s^−1^ in focused X-ray beams that are all considerably smaller than 10 µm.

The advent of XFELs has opened up new possibilities for structural biology studies. The extremely short and intense X-ray pulses not only allow dynamic studies with temporal resolution in the femtosecond range, but also mitigate radiation damage during room-temperature studies, since the exposure time of a single X-ray pulse is shorter than the time required for radiation damage to manifest itself on the atomic scale (Neutze *et al.*, 2000 ▸). However, in order to exploit these capabilities fully, new instrumentation and methods needed to be developed, which are now known as serial crystallography. A significant proportion of these developments were carried out at DESY within large international collaborations.

In the next section of this paper, we describe the features and capabilities of DESY’s P11 beamline for macromolecular X-ray crystallography. Section 3 covers the developments of serial crystallography at XFELs, in particular at the European XFEL (EuXFEL), and at synchrotron radiation sources. Section 4 describes the scientific environment on the DESY and EuXFEL campuses regarding structural biology activities.

## 2. Macromolecular crystallography beamline P11 at PETRA III

### 2.1. Beamline hardware

**Figure 1:**
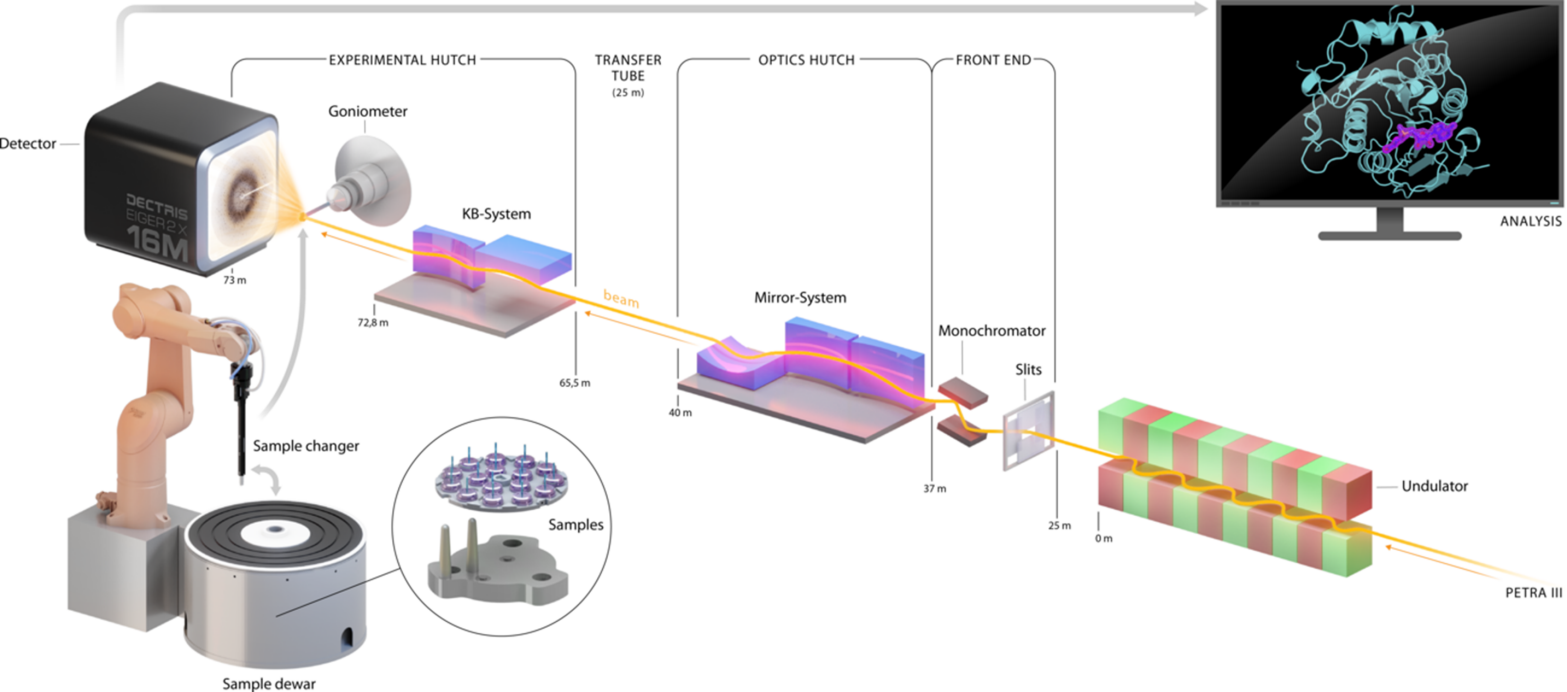
Schematic overview of the DESY-operated beamline P11 at PETRA III.

The DESY-operated beamline P11 at PETRA III (Burkhardt *et al.*, 2016 ▸; Fig. 1 ▸) is a high-throughput macromolecular crystallography beamline, in operation since January 2013. Initially designed for two techniques, X-ray microscopy and diffraction, the beamline is now mainly dedicated to crystallography. The instrument can be operated between 5.5 and 30 keV and offers variable beam sizes, from 200 µm × 200 µm to 9 µm × 4 µm (h × v) with a maximum flux of 10^13^ photons s^−1^. An undulator of 2 m in length serves as the P11 X-ray source. It has a minimum gap of 9.5 mm and a magnetic period of 31.4 mm with an r.m.s. source size of 141 µm × 5 µm and an r.m.s. divergence of 8.6 µrad × 5.2 µrad in the horizontal and vertical directions, respectively. After passing through power slits, the X-ray beam is monochromated by a double-crystal monochromator (FMB Oxford Ltd, Oxford, UK) with two possible pairs of crystals, Si(111) and Si(311). After monochromatization, the beam is reflected by two horizontal mirrors, deflecting by 5 mrad each, in order to give sufficient separation from the P12 beam produced in the same straight section, and a third vertical mirror. This Kirkpatrick–Baez (KB) system (Kirkpatrick & Baez, 1948 ▸), located 40 m from the source, generates a secondary source from which the beam is refocused to the sample position by another pair of KB mirrors located 72 m from the source (Fig. 1 ▸). The curvature of the mirrors can be dynamically adjusted by piezo-motors to change the beam size at the sample position. All mirrors are made of silicon and coated with Pd/Rh and Pt for efficient suppression of higher harmonics over the entire energy range.

The crystallography endstation, including an in-house designed single-axis high-precision diffractometer, various beam-shaping elements and the sample changer, is fully described by Burkhardt *et al.* (2016 ▸). The beamline operates with unipucks. Its sample changer Dewar has a capacity of 23 pucks (*i.e.* 368 sample mounts) and the sample cycle time is about 40 s. An EIGER2 X 16M detector (Dectris Ltd, Baden, Switzerland) replaced a PILATUS 6M-F detector in 2021 and increased the beamline throughput by speeding up data collection from a maximum frame rate of 40 Hz to 130 Hz. The typical data collection time for well diffracting crystals is about 18 s, without counting the time required to change the sample.

### 2.2. Beamline software

In conjunction with the recent detector upgrade, the automatic processing of the beamline data was migrated from a local computational cluster to the DESY Maxwell High-Performance Cluster for parallel and multi-threaded applications managed by a SLURM scheduler.

The diffraction data are automatically processed with *XDSAPP* (Sparta *et al.*, 2016 ▸) and upon demand with *Autoproc* (Vonrhein *et al.*, 2011 ▸); auto-processing jobs are triggered at dedicated computational nodes of the beamline via an in-house developed data acquisition program. This graphical user interface is now being replaced by *MXCuBE* (Oscarsson *et al.*, 2019 ▸), in order to harmonize P11 with the neighbouring MX beamlines P13 and P14 and to engage fully with the *MXCuBE* and *ISPyB* (Delagenière *et al.*, 2011 ▸) collaborations. The deployment of *EDNA* (Incardona *et al.*, 2009 ▸) and *Dozor* (Melnikov *et al.*, 2018 ▸), which are required for sample characterization and grid scans, respectively, in *MXCuBE*, will also be realized on the DESY Maxwell cluster. Its current status is described by Gruzinov *et al.* (2025 ▸). *ISPyB* is currently being implemented with *EXI* as the user interface.

### 2.3. User access schemes and operation modes

A scheme for remote access, implemented in 2020, is now the main operation mode of the beamline, with on-site access used mainly by local groups or for serial crystallography. Remote users collect data via a *FastX* (StarNet Communications, Santa Clara, California, USA) connection in a web browser. As a consequence, the number of sample shipments has increased significantly, and to cope with this situation a Dewar hotel (Hänel Büro- und Lagersysteme GmbH, Bad Friedrichshall, Germany) has recently been installed and is operated jointly by the DESY and EMBL MX beamlines for storage of the dry shippers.

To complement the high throughput of the beamline, an efficient fragment screening pipeline has recently been established, expanding the support towards sample preparation and data analysis. Samples are prepared using the crystallization platform of the Sample Preparation and Characterization Facility at EMBL Hamburg and crystal growth can be followed using the *CRIMS* management system (Cornaciu *et al.*, 2021 ▸). Fragment soaking and crystal harvesting can be performed by acoustic ejection using the Echo 550 (Collins *et al.*, 2017 ▸) and the motorized interactive microscope stage Crystal Shifter (Oxford Laboratory Technologies Ltd) (Wright *et al.*, 2021 ▸), respectively. Sample information is transferred electronically from records of crystallization, soaking and fishing the crystals to the beamline. After data collection and auto-processing, the structure solution and fragment binding analysis are performed using scripts running *Dimple* (Wojdyr *et al.*, 2013 ▸) and *PanDDA* (Pearce *et al.*, 2017 ▸) on the Maxwell cluster.

An alternative setup on the beamline, making use of the high flux and small focus of the X-ray beam as well as the fast detector, is serial synchrotron crystallography (SSX) using a TapeDrive system (Zielinski *et al.*, 2022 ▸). Here, a stream of crystalline slurry is carried across the beam on a tape that moves at a constant velocity perpendicular to the beam axis. This slurry is applied to the tape just upstream of the beam intersection through a stationary nozzle. The sample is pumped at constant pressure through the nozzle, which can accommodate an additional channel for mixing with a substrate or another substance of interest. The exposure time, set by the time taken for a crystal to traverse the beam focus, can be further reduced to 3.5 ms with the use of an X-ray chopper. Diffraction data are auto-processed using *CrystFEL* (White *et al.*, 2012 ▸; White *et al.*, 2025 ▸).

The industrial use of P11 has been steadily increasing, both in the number of industrial shifts and in the number of industrial customers, from 1.5% in 2016, when industrial use was introduced, to nearly 11% of the total allocated beam time in 2023. The represented entities include contract research organizations and pharmaceutical companies, with half of the experiments conducted on the beamline outsourced as mail-in experiments.

A pilot project of a new rolling access scheme was implemented on several beamlines at PETRA III, including beamline P11, in the spring of 2024. In March 2024, the beamline switched from the regular to the rolling call for proposals as the only access mechanism. Rolling proposals can be submitted at any time, are evaluated without deadlines, and speed up the process from beam time application to experiment by several months in the best case. The block allocation proposals (BAGs) and long-term proposals (LTPs) that were still in progress based on previous calls retained their access until the end of their terms.

Eventually, the new adaptive access model will include a project-based access with the possible use of several beamlines, laboratories and facilities on the DESY campus. Such an approach is already implemented on beamline P11 with the aforementioned fragment screening pipeline, a classic example of the use of additional laboratories and processes, as well as of the delegation of parts of the experiment to the beamline staff. Computational approaches are currently under development to coordinate the use of multiple beamlines and laboratories and to combine the results. Here we aim to merge existing open-access software solutions connected with our user portal and computational cluster, such as an electronic laboratory notebook and metadata catalogue.

### 2.4. Science programme

From first light on the beamline in 2012 to November 2024, a total of 762 structures resulting from data collected on P11 have been deposited in the Protein Data Bank, mainly coming from standard single-crystal crystallography. In recent years, more than 80% of the beam time has been dedicated to regular rotation crystallography of protein and RNA samples, 10% to samples and method development for serial crystallography, 5% to imaging techniques, and 5% to regular rotation crystallography of non-biological samples requiring a protein crystallography-like setup, such as for supramolecular complexes. The user community is predominantly from Germany, followed by neighbouring countries the Netherlands, Poland and Austria. More than 25 different countries are represented within the users’ home institutions. In 2022, the beamline hosted over 1000 user visits through the year and over 250 unique users. To date, 362 publications have resulted from experiments on P11. Examples include work on enzym­ology, bioengineering (Fan *et al.*, 2023 ▸), RNA research (Mieczkowski *et al.*, 2021 ▸), fragment screening (Wang *et al.*, 2022 ▸), drug discovery (Visser *et al.*, 2023 ▸, Qiao *et al.*, 2021 ▸), large drug screening campaigns (Günther *et al.*, 2021 ▸), work on SARS-CoV-2 (Reinke *et al.*, 2024 ▸; Reinke *et al.*, 2023 ▸), experimental phasing, serial crystallography (Prester *et al.*, 2024 ▸; Henkel *et al.*, 2023 ▸), and X-ray optics (Bajt *et al.*, 2018 ▸; Dresselhaus *et al.*, 2024 ▸) and imaging (Zhang *et al.*, 2024 ▸).

### 2.5. Future developments

The PETRA III facility at DESY is proposed to be upgraded to the world’s most brilliant synchrotron radiation source, PETRA IV. PETRA IV is expected to operate at 6 GeV and up to 200 mA beam current with an emittance of 12 pm rad × 12 pm rad compared with the current 1300 pm rad × 15 pm rad, in the horizontal and vertical directions, respectively. Depending on the photon energy, an increase in brilliance by about a factor of 100 up to 1000 is expected, which will be relevant for MX beamlines for the micrometre focal spot sizes mainly used for serial synchrotron crystallography. As part of this upgrade process the PETRA III instruments will also be upgraded, and a new beamline portfolio has been defined in close collaboration with the user community and the DESY scientific advisory boards. Also, for PETRA IV, the three MX beamlines will remain at their current locations in sectors 8 and 9 of the Max von Laue experimental hall, together with the bio-SAXS beamline. These beamlines will form a structural biology ‘village’ to provide a unified user experience. Several preparatory projects are already ongoing to streamline the operations, *e.g.* by using the same graphical user interfaces and sharing the same sample storage. The new beamline design is currently in the conceptual design phase. The DESY MX beamline P11 will continue to operate as a dedicated high-throughput instrument with an optional unattended data collection mode. The experimental portfolio will be extended to include κ-goniometry and a top-hat beam for high-precision intensity measurements. The beamline instrumentation and the associated computational and data storage capabilities will be upgraded to cope with even faster experiments, and the sample changer capacity will be increased to handle a larger number of samples.

## 3. Serial crystallography

### 3.1. Introduction

Originally, serial crystallography (SX) (Henkel & Oberthür, 2024 ▸) was an idea first proposed by John Spence and colleagues at Arizona State University (ASU) in the USA for single-particle electron diffraction in liquid helium droplets (Spence & Doak, 2004 ▸). However, what is understood as SX today was pioneered by a collaboration led by scientists from DESY. The first proof-of-principle experiment to outrun radiation damage and to show that useful diffraction can be obtained before destruction of the sample by an intense femtosecond-duration X-ray pulse from an FEL was carried out on FLASH at DESY (Chapman *et al.*, 2006 ▸). This was soon followed by the first experiment (Chapman *et al.*, 2011 ▸) for serial crystallographic protein structure determination using a hard XFEL, conducted at the Linac Coherent Light Source (LCLS) (Emma *et al.*, 2010 ▸) at the SLAC National Accelerator Laboratory (Menlo Park, California, USA). Today there are experimental stations for serial femtosecond crystallography (SFX) at all existing XFEL facilities and the method is used to unravel structural dynamics of biological macromolecules on ultra-short time scales (Brändén & Neutze, 2021 ▸).

### 3.2. Software development

There were initially two major challenges in developing SFX: analysis software and sample delivery. In both cases, a collaboration between the Spence team at ASU and the Coherent Imaging Group at DESY was critical to overcoming these challenges, ultimately paving the way for today’s success of the method. In data processing, existing software relied on the condition that a rotation series of diffraction images from the same crystal had been recorded, and information on the relative orientation of the crystal was essential to evaluate diffraction images. In SFX, each crystal is randomly orientated and probed only once by an exposure that freezes its movement in time. Existing software was adapted and used with partial success to index the resulting individual diffraction images, but the integration, merging and scaling of the diffraction peak intensities required a new approach. Initial concepts were developed to overcome this hurdle (Kirian *et al.*, 2010 ▸; Kirian *et al.*, 2011 ▸) and could be used to process and analyse the recorded datasets, resulting in merged and reduced *hkl*–intensity files that could be used for subsequent phasing and crystallographic refinement and model building. These efforts were then combined in the software suite *CrystFEL* (White *et al.*, 2012 ▸), which is still under constant development at DESY (White, 2019 ▸; White *et al.*, 2016 ▸), and is to date the most commonly used software for data analysis of both SFX and SSX experiments.

### 3.3. Sample delivery

Sample delivery for SFX was initially carried out using liquid jets generated by gas dynamic virtual nozzles (GDVNs), developed for measurement of diffraction of microscopic crystals with a continuous (CW) or pulsed electron or X-ray beam (DePonte *et al.*, 2008 ▸). These GDVNs were compatible with the high-vacuum environment of the first experimental stations for SFX (AMO and CXI at LCLS). They would recover from the explosion of the jet caused by the interaction of the intense X-ray pulse with the liquid column (Stan *et al.*, 2016 ▸), in time for the next X-ray pulse. The relatively high sample consumption of these liquid jets motivated research to either improve or replace liquid jets for sample delivery.

High-viscosity extrusion (HVE) systems, using a highly viscous matrix for sample delivery, were developed (Weierstall *et al.*, 2014 ▸) for membrane protein SFX (Kang *et al.*, 2015 ▸; Liu *et al.*, 2013 ▸). These extrusions offer a significantly lower sample consumption and a compatibility with crystals grown in lipidic cubic phase (LCP), as this was the first matrix used for this approach. The disadvantage of this type of sample delivery is the larger diameter of the column of medium and thus higher background scattering from the interaction with the high-intensity X-rays. Nowadays, other materials such as cellulose, agarose or grease are also used as viscous matrices for HVE injection, and this method is widely used at XFELs such as SwissFEL or SACLA and many synchrotron beamlines.

Another derivative of liquid jet injection is the microfluidic electrokinetic sample holder (MESH) (Sierra *et al.*, 2012 ▸) and its further development the concentric MESH injector (CoMESH) (Sierra *et al.*, 2016 ▸). Here, a voltage applied to the sample and injection nozzle towards a grounded counter-electrode drives the sample instead of pressure, resulting in lower sample consumption than with a GDVN.

Improvements to make GDVN injection more reliable and with lower sample consumption were introduced by teams at DESY. One weak point of nozzles was that their fabrication and assembly were carried out manually and thus nozzle-to-nozzle variations were often large. Using ceramic micro-injection moulding, the outer part of a GDVN could be made in an automated and reproducible fashion (Beyerlein *et al.*, 2015 ▸). Double-flow focusing nozzles (DFFNs), a variation of GDVNs in which jetting is achieved by interaction of an outer sheath liquid (usually ethanol or propan-2-ol) with the focusing gas (usually He), provided further advantages. The sample is injected into the sheath liquid and forms a jet within the jet. Since jetting is achieved by the sheath liquid, the sample flow rate can be much lower than in a GDVN. Furthermore, the sheath liquid stabilizes the jet and reduces the risk of ice formation in a vacuum (Oberthuer *et al.*, 2017 ▸).

Again in a collaboration between ASU and DESY, the possibilities of producing GDVNs by means of two-photon stereolithography 3D printing with nano-precision were explored (Nelson *et al.*, 2016 ▸; Knoška *et al.*, 2020 ▸). Three-dimensionally printed GDVNs and DFFNs are now the standard for liquid jet sample delivery for SFX. Early on, DESY was involved in the development of fixed targets as an alternative to liquid jet sample delivery for SFX (Roedig *et al.*, 2015 ▸; Roedig *et al.*, 2016 ▸; Roedig *et al.*, 2017 ▸). These fixed target chips use minimal amounts of sample and are designed for low background scattering.

### 3.4. SFX at EuXFEL

Many of the groups involved in the first serial crystallography experiments at the LCLS were instrumental in setting up the SFX User Consortium at the European XFEL, located in Schenefeld, close to Hamburg (Decking *et al.*, 2020 ▸). The user consortium organized and contributed funding for a serial femtosecond crystallography programme to be operated alongside the initially planned diffraction imaging programme to form the Single Particle, Clusters and Biomolecules & Serial Femtosecond Crystallography (SPB/SFX) instrument^3^ (Mancuso *et al.*, 2019 ▸; Mills *et al.*, 2020 ▸). The first user experiment at EuXFEL was conducted by an international consortium led by DESY and established megahertz SFX (MHz-SFX) utilizing the unique pulse pattern of that facility (Wiedorn, Awel *et al.*, 2018 ▸; Wiedorn, Oberthur *et al.*, 2018 ▸; Yefanov *et al.*, 2019 ▸). EuXFEL uses *CrystFEL* as a standard for data processing (Sobolev *et al.*, 2024 ▸) and is further developing DFFN, GDVN and microfluidic mixers based on DESY designs (Vakili *et al.*, 2022 ▸). A DESY-led team used the nanofocus capabilities of SPB/SFX in combination with 3D-printed DFFNs (Knoška *et al.*, 2020 ▸) to solve the structure of the bacterial insecticidal protein Tpp49Aa1 from native sub-micrometre crystals (Williamson *et al.*, 2023 ▸).

**Figure 2:**
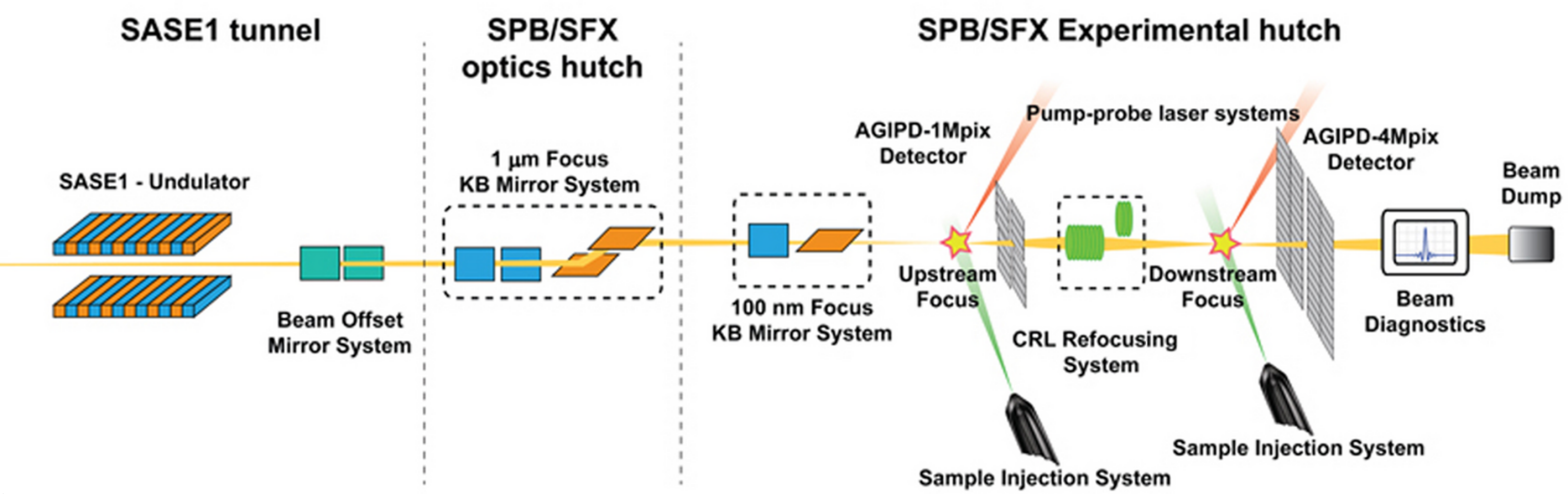
Schematic overview of the SFB/SFX experimental station at the European XFEL. Beam transport is from left to right, showing the interaction region with the AGIPD 1M on the left and the (still under construction) interaction region with the AGIPD 4M on the right of the image.

The SFX User Consortium contribution also includes a dedicated endstation for high-resolution serial crystallography on SPB/SFX, currently in the final stages of development and construction. The endstation will employ a 4 Megapixel version of the adaptive gain integrating pixel detector (AGIPD), developed by an international collaboration led by the DESY photon detector group and based on experiences of the 1 Megapixel detector already installed on the initial SPB/SFX endstation (Allahgholi *et al.*, 2019 ▸; Sztuk-Dambietz *et al.*, 2024 ▸). A schematic overview of the layout of the SPB/SFX instrument with both endstations installed is shown in Fig. 2 ▸. The AGIPD can capture up to 352 pulses at the 4.5 MHz train pulse rate of the European XFEL with individual pixel gain adjustment to measure a wide dynamic range in Bragg peak intensities. Optical laser systems, developed to complement the X-ray pulse delivery pattern, allow time-resolved crystallography experiments down to femtosecond time resolution (Koliyadu *et al.*, 2022 ▸). Reactions not initiated by light can be studied via mix-and-inject techniques (Pandey *et al.*, 2021 ▸) and via the release of reactants through photo-cage compounds (de Wijn *et al.*, 2022 ▸).

The SPB/SFX endstation continues to host innovative experiments to maximize the use of the megahertz-rate pulses and increase the efficiency of sample use, with several encouraging developments. Segmented-flow jets (Doppler *et al.*, 2024 ▸) inject sample-containing segments into a continuously flowing buffer jet. Spinning-disc sample delivery is an analogue of TapeDrive systems for continuous or low repetition rate sources, with a variety of sample-dependent material and enclosure options. Beam sweeping (Chapman *et al.*, 2017 ▸), developed in collaboration with Alke Meents and colleagues at DESY, uses a rotating mirror system to deflect the incoming megahertz-rate X-ray pulses into the sample plane, reducing the sample refresh rate required.

### 3.5. SX at synchrotron radiation sources

Early in the first months of user operations, SX experiments were conducted on beamline P11 in 2013 by a team from the Coherent Imaging Group at DESY. For this, protein crystals were flowed in random orientation in a thin glass capillary through the X-ray focus. The high X-ray intensity on P11, the state-of-the-art Pilatus 6M detector (at the time) and the data processing tools of the DESY team enabled for the first time serial crystallography data collection at a synchrotron (Stellato *et al.*, 2014 ▸). The method is now commonly referred to as serial synchrotron crystallography (SSX). This ground-breaking experiment inspired other teams, for example at the Swiss Light Source (SLS) (Botha *et al.*, 2015 ▸) and the European Synchrotron Radiation Facility (ESRF) (Nogly *et al.*, 2015 ▸). The first SSX experiments were carried out by Perry *et al.* (2014 ▸). Also, at DESY, a team established SSX using polychromatic X-rays (Meents *et al.*, 2017 ▸) and developed special indexing algorithms to improve data processing further (Gevorkov *et al.*, 2020 ▸). Nowadays, SSX has been established in various ways at most synchrotron facilities around the globe. With ID29 at ESRF, MicroMAX at MAXIV and T-REXX at PETRA III, there are three experimental stations in operation predominantly dedicated to SSX (Henkel & Oberthür, 2024 ▸). At PETRA III, SSX can be conducted on T-REXX and P14 (both operated by EMBL), as well as on P11 (operated by DESY). On T-REXX, various sample environments can be used, even though it is mainly used for fixed-target SSX (Mehrabi, Schulz, Agthe *et al.*, 2019 ▸; Mehrabi, Schulz, Dsouza *et al.*, 2019 ▸; Bjelčić *et al.*, 2024 ▸). On P14, serial helical line scans (Schönherr *et al.*, 2024 ▸) and HVE injection can be used for SSX (Kovalev *et al.*, 2023 ▸). For P11, the aforementioned TapeDrive system for sample delivery was developed at DESY (Beyerlein *et al.*, 2017 ▸; Zielinski *et al.*, 2022 ▸; Henkel *et al.*, 2023 ▸). This combines advantages from fixed-target SSX (low sample consumption) with those from liquid jets (continuous data collection). It can also be used for mix-and-inject time-resolved SSX (Beyerlein *et al.*, 2017 ▸; Prester *et al.*, 2024 ▸) and rapid just-in-time crystallization (Henkel *et al.*, 2023 ▸). Additionally, HVE injection is possible on P11 (Botha *et al.*, 2018 ▸), although this is not currently available to users.

## 4. Scientific campus environment

The joint campus of DESY and the University of Hamburg forms the core of the Science City Hamburg Bahrenfeld (SCHB) (https://www.sciencecity.hamburg/en). In addition to the large-scale photon science user facilities mentioned above, a number of interdisciplinary science centres have been established on campus in conjunction with partner institutions during the last decade. These centres make strong contributions to the use and further development of the large-scale photon science facilities. Historically, the first was the Centre for Free-Electron Laser research (CFEL, https://www.cfel.de), a collaboration between DESY, the Max Planck Society and the University of Hamburg. CFEL’s main aim is to target unresolved scientific challenges on ultra short timescales and with extremely high photon fluxes. The DESY CFEL coherent imaging group is instrumental in the development of the SFX and SSX techniques, both in terms of hardware, methods and software developments, and in the pursuit of a biology-related research agenda. A collaboration team housed in the CFEL building and led by the DESY photon science detector group developed the adaptive gain integrated pixel detector (AGIPD) technology (Allahgholi *et al.*, 2019 ▸) capable of recording a burst of 2D diffraction images at a frame rate of 5 MHz for up to 352 exposures. This technology is the key to exploit the EuXFEL bunch structure for SFX experiments. The Centre for Structural Systems Biology (CSSB) on the DESY campus (https://www.cssb-hamburg.de) is a joint centre of nine partner institutions from Northern Germany including DESY and the EMBL, with the goal of elucidating the molecular mechanism of infections. Complementary to the photon science facilities operated by DESY, EMBL and EuXFEL, the CSSB operates analytical facilities such as state-of-the-art confocal light microscopes and four cryo-electron microscopes for structural biology investigations. Two of these high-end microscopes are also used for cryo-electron microscopy tomography experiments. The newest research centre on the SCHB campus is the Hamburg Advanced Research Centre for Bioorganic Chemistry (HARBOR, https://www.cui.uni-hamburg.de/harbor.html) of the University of Hamburg. The project has sprung directly from research at the Hamburg Centre for Ultrafast Imaging and closes the gap between physics and life sciences research on campus. Scientists from HARBOR are heavily involved in the construction, operation and use of the T-REXX station on P14 at PETRA III.

## 5. Summary

Even though new AI-based structure prediction algorithms (Jumper *et al.*, 2021 ▸) and the most recent achievements in cryo-electron microscopy (Kuehlbrandt, 2014 ▸) have led to a paradigm shift in structural biology, synchrotron radiation macromolecular X-ray crystallography is still a heavily requested experimental technique for the accurate and high-resolution determination of macromolecular structures, fragment screening or kinetic studies, just to name some of the requested applications. As a complement to synchrotron radiation sources, experiments at XFELs allow the SFX technique to be used to study extremely small crystals, which is not possible at synchrotron radiation sources, and, more importantly, to study the dynamics and function over a wide range of time scales down to the femtosecond regime. Synchrotron radiation-based biological SAXS experiments are also of great value for life science studies of larger assemblies, as has been demonstrated during the past COVID-19 pandemic [*e.g.* Graewert *et al.* (2023 ▸)]. Thus, the photon science facilities at DESY and on the EuXFEL campus are well placed to serve a large national and international user community in all fields of science in general, and in structural biology in particular, for experiments on all relevant length and time scales. Staff at these facilities interact heavily with various campus partners, triggering new technical and method­ological developments, as well as making contributions to the solutions to basic scientific questions and concrete problems of societal relevance. For the medium-term future, DESY proposes to upgrade its PETRA III storage ring to PETRA IV to provide diffraction-limited radiation in the tender X-ray regime. All techniques that require an extremely small and intense X-ray focus, like SSX or coherent X-ray imaging techniques aiming for nanometre spatial resolution, will dramatically benefit from the improved beam performance and will push the limits of applicability of these techniques towards high resolution in time and space.

## Footnotes

1 https://photon-science.desy.de/facilities/petra_iii/beamlines/index_eng.html.

2 https://www.embl.org/services-facilities/hamburg/.

3 https://www.xfel.eu/facility/instruments/spb_sfx/index_eng.html.
