# Present and future structural biology activities at DESY and the European XFEL. Erratum

**DOI:** 10.1107/S1600577525003728

**Published:** 2025-04-25

**Authors:** Dominik Oberthür, Johanna Hakanpää, Spyros Chatziefthymiou, Guilllaume Pompidor, Richard Bean, Henry N. Chapman, Edgar Weckert

**Affiliations:** a Deutsches Elektronen-Synchrotron DESY Notkestrasse 85 22607Hamburg Germany; b European XFEL Holzkoppel 4 22869Schenefeld Germany; c Centre for Ultrafast Imaging, Luruper Chausee 149, 22761Hamburg, Germany; d Department of Physics University of Hamburg Luruper Chausee 149 22761Hamburg Germany

**Keywords:** structural biology, macromolecular crystallography, serial crystallography, FEL and synchrotron radiation

## Abstract

A correction in the paper by Oberthür *et al.* [(2025). *J. Synchrotron Rad.***32**, 474–485] is made.

There is a typographical error in the article by Oberthür *et al.* (2025 ▸). In the second paragraph of Section 1 it states that the circumference of PETRA III is 2304 km – the correct value is 2.304 km.
